# Non-invasive Investigation of Human Hippocampal Rhythms Using Magnetoencephalography: A Review

**DOI:** 10.3389/fnins.2018.00273

**Published:** 2018-04-26

**Authors:** Yi Pu, Douglas O. Cheyne, Brian R. Cornwell, Blake W. Johnson

**Affiliations:** ^1^ARC Centre of Excellence in Cognition and its Disorders, Macquarie University, Sydney, NSW, Australia; ^2^Department of Cognitive Science, Macquarie University, Sydney, NSW, Australia; ^3^Program in Neurosciences and Mental Health, Hospital for Sick Children Research Institute, Toronto, ON, Canada; ^4^Department of Medical Imaging, University of Toronto, Toronto, ON, Canada; ^5^Brain and Psychological Sciences Research Centre, Swinburne University of Technology, Melbourne, VIC, Australia

**Keywords:** magnetoencephalography (MEG), hippocampus, deep source imaging, simulation and empirical evidence, review

## Abstract

Hippocampal rhythms are believed to support crucial cognitive processes including memory, navigation, and language. Due to the location of the hippocampus deep in the brain, studying hippocampal rhythms using non-invasive magnetoencephalography (MEG) recordings has generally been assumed to be methodologically challenging. However, with the advent of whole-head MEG systems in the 1990s and development of advanced source localization techniques, simulation and empirical studies have provided evidence that human hippocampal signals can be sensed by MEG and reliably reconstructed by source localization algorithms. This paper systematically reviews simulation studies and empirical evidence of the current capacities and limitations of MEG “deep source imaging” of the human hippocampus. Overall, these studies confirm that MEG provides a unique avenue to investigate human hippocampal rhythms in cognition, and can bridge the gap between animal studies and human hippocampal research, as well as elucidate the functional role and the behavioral correlates of human hippocampal oscillations.

## Introduction

The hippocampus is an important brain region for various cognitive processes, including spatial navigation (O'Keefe and Nadel, 1978; Buzsaki and Moser, 2013), memory (Horner and Doeller, 2017), and language comprehension (Piai et al., 2016). Neuronal oscillations are believed to be important mechanisms for these processes (Colgin, 2016) and it is thus of great importance to understand the functions of hippocampal rhythms. At the present time, there are several different methods available to investigate the function of the human hippocampus. fMRI is frequently used in experimental studies of hippocampal activation in healthy humans; however due to its limited temporal resolution (Buckner and Logan, 2001), the frequency specificity and time course of rhythmic neuronal activity cannot be resolved with this technique. The scalp electroencephalogram (EEG) provides high temporal resolution on a timescale of milliseconds. However, source reconstruction of the EEG is complicated by the fact that electrical signals are vulnerable to distortions by skull, skin, and cerebrospinal fluid (CSF) (Nunez and Srinavasan, 2006; Lopes da Silva, 2010; Cohen, 2017). Intracranial EEG (iEEG) provides both excellent spatial and temporal resolution, but it depends on very limited opportunities to obtain recordings from surgical patients. In addition, electrode coverage and spatial sampling is sparse, with limited ability to examine interactions between the hippocampus and other brain areas supporting memory function.

Magnetoencephalography (MEG) measures the brain magnetic fields induced by synchronous neuronal populations, with superconducting sensors in a heavily shielded room (Cohen, 1972). Compared to scalp-recorded EEG, MEG has an advantage in identifying brain currents giving rise to the signals (Hari et al., 2000) recorded from MEG sensors outside the brain, because the skull, skin, and CSF are almost transparent to magnetic fields. This advantage allows MEG to contribute to comprehending and exploiting both regional and large-scale neural dynamics by clarifying the nature of spontaneous and event-related brain activities and by the elucidation of the mechanisms underlying inter-regional connectivity (for a recent review see Baillet, 2017). Due to its non-invasive nature, MEG may provide an avenue to study the function of neuronal dynamics of the human hippocampus by routine experimentation. Accordingly, it would play an important role in connecting the human data with animal and computational models of electrophysiology in health and disease (Baillet, 2017). However, whether MEG can reliably detect hippocampal signals has been a topic of debate, due to the following considerations. First, magnetic signals decay rapidly with distance, so signals from the hippocampus are thus assumed to be strongly attenuated relative to signals from the neocortex (Hämäläinen et al., 1993; Hillebrand and Barnes, 2002). Second, some widely used source localization techniques such as minimum norm estimation (MNE) are strongly biased toward the neocortical surface and away from deep brain regions (Attal and Schwartz, 2013). Third, some studies (Mikuni et al., 1997; Oishi et al., 2002; Wennberg and Cheyne, 2014) have reported variable and limited ability of MEG to detect interictal spiking in the hippocampus and medial temporal region of epileptic patients observable with intracranial electrodes or electrocorticography (ECoG) grids. Fourth, the folded nature of the hippocampal morphology may lead to signal cancelation (Mikuni et al., 1997).

However, with the advent of whole-brain MEG systems in the 1990s (Ahonen et al., 1993) and mathematical and computational advances in MEG source localization (such as beamforming techniques), a number of laboratories have reported detection of hippocampal signals with MEG (e.g., Tesche et al., 1996; Nishitani et al., 1998; Tesche and Karhu, 1999; Cornwell et al., 2008; Moses et al., 2009; Backus et al., 2016; Pu et al., 2017). A series of numerical simulation studies (e.g., Stephen et al., 2005; Attal and Schwartz, 2013; Meyer et al., 2017b) have been carried out to systematically investigate the feasibility of and limits on MEG measurements of hippocampal activity under known conditions of source strength and depth for different magnetic sensor designs. The present review aims to synthesize the findings of the simulation and empirical studies of MEG measurements of hippocampal activity to date.

## Anatomy of the hippocampus

The hippocampus is one of several related brain regions that together constitute a functional system called the hippocampal formation (Amaral and Lavenex, 2006). The constituent areas include the dentate gyrus, hippocampus proper, subicular complex (subiculum, presubiculum, and parasubiculum), and entorhinal cortex (Insausti, 1993). The hippocampus proper has three subfields: CA1, CA2, and CA3 (CA is short for *cornu ammonis*, meaning “Ammon's horn,” which refers to the ram-headed god Amun of Egyptian mythology). Some researchers further subdivide CA3 into CA3 and CA4 regions. The basic morphology of the mammalian hippocampus proper is an elongated, curved and rod-like structure (Insausti, 1993). The hippocampus proper consists of one layer of principal neurons (e.g., pyramidal neurons) (Forster et al., 2006), which are neatly arranged in parallel with the dendrites aligned perpendicularly to the surface of the hippocampus proper. Due to the geometric configuration of pyramidal neurons with the dendrites facing one direction and the soma another (i.e., an open-field configuration), the electrical fields from such cells can extend over long distances and can induce substantial ionic flow in the extracelluar medium (Lorente de No, 1947). Theoretically, synchronized activation of cells with an open-field configuration could produce signals measurable at a distance by MEG and EEG (Murakami and Okada, 2006). Empirically, using hippocampal tissue preparations, Okada et al. (1997) showed that the magnetic evoked field is directly related to the dipolar intracellular currents of the pyramidal cells. They further estimated that the current dipole moment density (i.e., the electric field strength per unit volume) of the hippocampal tissue is larger than that which can be produced by the neocortex, because the neocortical tissue has more layers and a more complicated geometry than the hippocampus, with more potential for cancellation of currents across layers. Thus, the hippocampus may generate stronger signals than neocortex, compensating for the greater distance from the MEG sensor array (Chupin et al., 2002; Attal et al., 2007).

## MEG

**Figure 1:**
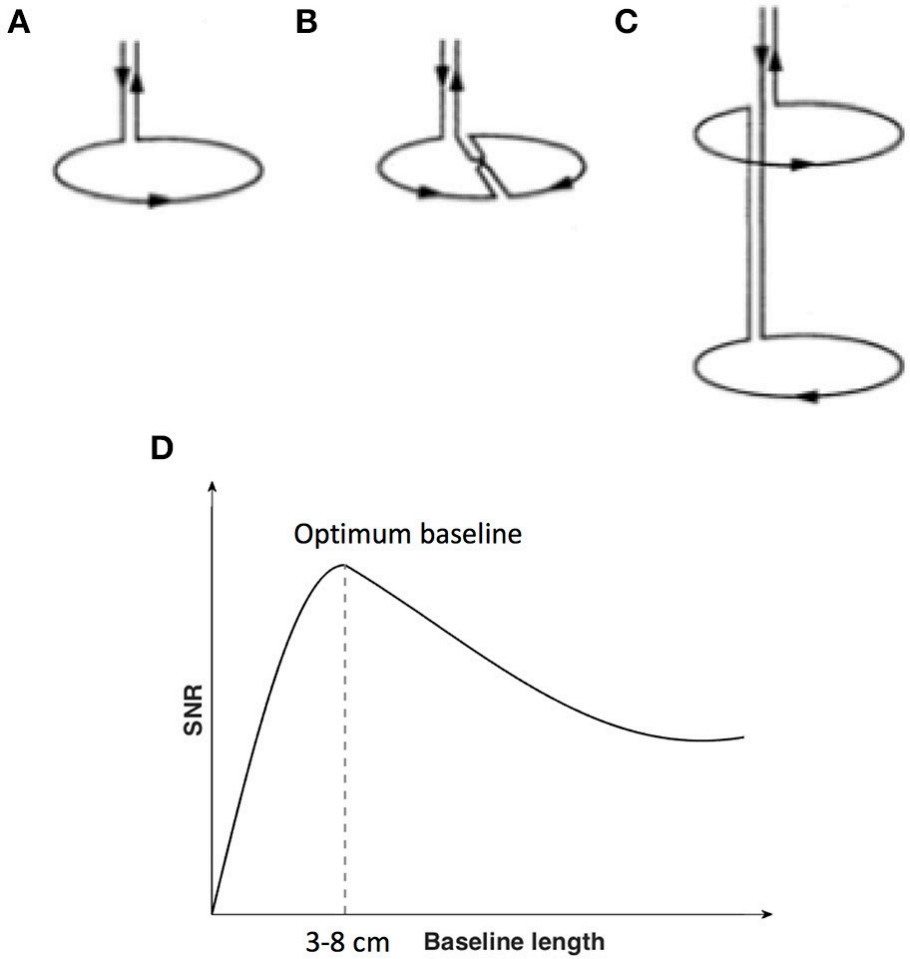
Flux transformer (pick up coils). **(A)** Magnetometer; **(B)** First-order planar gradiometer **(C)** First-order axial gradiometer. **(D)** Signal-to-noise ratio (y-axis with arbitrary units) of MEG sensors as a function of the length of the baseline of the flux transformer pick up coils (x-axis). **(A–C)** is reproduced with permission from Hämäläinen et al. (1993). **(D)** is adapted from Vrba and Robinson (2001).

MEG is a technique for measurement of human brain function via detection and interpretation of magnetic fields emanated from the brain, with millisecond temporal resolution (Cohen, 1968, 1972; Hämäläinen et al., 1993; Ioannides, 2006; Cheyne and Papanicolaou, 2017). Compared with Earth's magnetic field and urban magnetic noise, the magnetic field of the brain is about a factor of 1 million to 1 billion times smaller (Vrba and Robinson, 2001). To detect such small magnetic fields, highly sensitive detectors are needed in conjunction with noise reduction techniques. Current technology is based on the superconducting quantum interference device (SQUID) coupled with flux transformers (or pick up coils) bathed in cryogen, and contained within a magnetically shielded room (MSR) to increase the overall magnetic field sensitivity (Fagaly, 2006). One technical issue important to the detectability of deeper brain activity is the choice of MEG pick up coil design. The pickup coils can have various configurations and different commercial MEG systems employ different types (Table 1). There are three main types of coil configurations currently in use (Figures 1A–C): magnetometers with a single loop of wire; and axial gradiometers and planar gradiometers with two or more loops combined with opposite orientation and with a certain distance between the two coils or baseline. Gradiometers are used to improve rejection of environmental magnetic fields through signal cancellation, as these signals will have a similar but opposite amplitude at each coil, while brain sources close to the gradiometer will have a differential input on each of the coils.

**Table 1:** Commercial MEG systems currently in widespread use.

**MEG system**	**Pickup coil configuration**	**Baseline**	**Reference sensors**
CTF 151 (VSM/MISL) (Port Coquitlam, Canada)	151 first order axial gradiometers	5 cm	29 gradiometers
CTF 275 (VSM/MISL) (Port Coquitlam, Canada)	275 first order axial gradiometers	5 cm	29 gradiometers
KIT/Yokogawa (Kanazawa, Japan)	160 first order axial gradiometers	5 cm	Optional, gradiometers
Elekta-Neuromag 122 (Helsinki, Finland).	61 orthogonal pairs of first order planar gradiometers	1.4 –1.6 cm	None
Elekta-Neuromag 306 (Helsinki, Finland)	102 magnetometers + 102 orthogonal pairs of first order planar gradiometers	1.4 –1.6 cm	None
4D Neuroimaging 148 (San Diego, USA)	148 magnetometers	Infinite	23 magnetometers and gradiometers
4D Neuroimaging 248 (San Diego, USA)	248 magnetometers	Infinite	23 magnetometers and gradiometers

**Figure 2:**
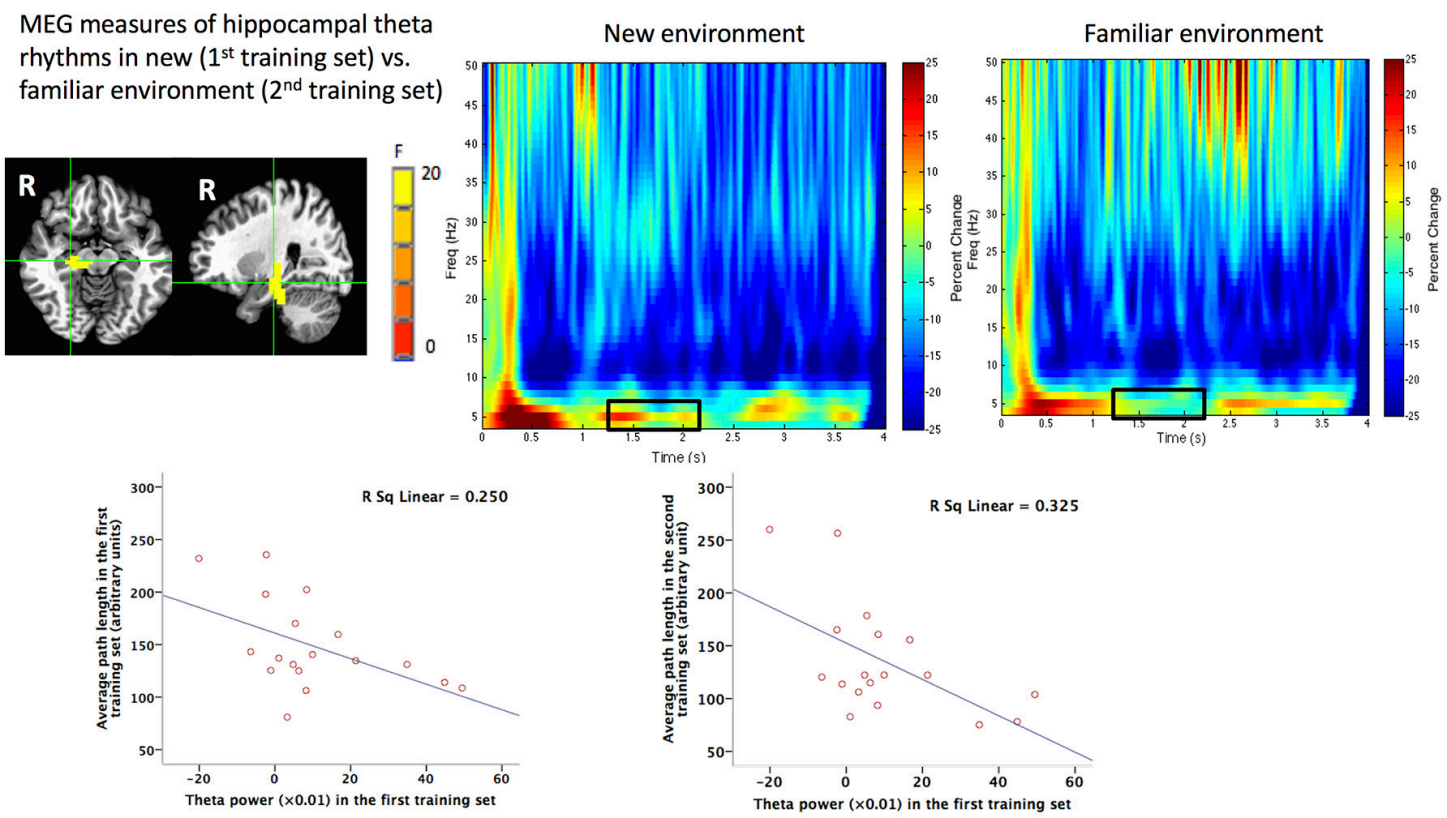
MEG measures of human hippocampal theta oscillations (4–8 Hz) during spatial navigation and its behavioral correlates. **Upper**: Group image (*N* = 18) of main effect of environmental novelty in the time window of 1.25–2.25 s during virtual spatial navigation in the right hippocampus revealed by beamforming analyses and the time frequency representations (TFRs) of the virtual sensor placed in the peak voxel of right hippocampus in new and familiar environment. The black squares on the TFRs indicate more theta power during 1.25–2.25 s in the new (1st training set) vs. familiar (2nd training set) environment. **Lower**: Theta power in the first training set (new environment) in the right hippocampal region shown in the upper left panel plotted against averaged path lengths (arbitrary units) in the first or second training set in the navigation task. This figure is reproduced with permission from Pu et al. (2017).

**Figure 3:**
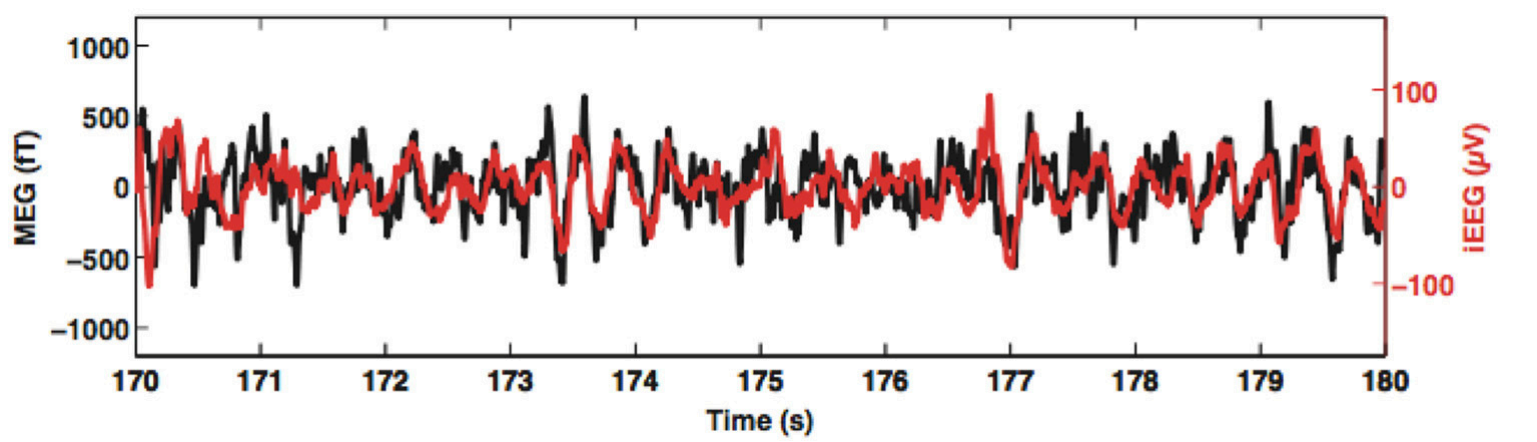
Simultaneously recorded magnetoencephalogram (MEG; black trace) and hippocampus depth electroencephalogram (EEG; red trace) from a pre-surgery patient. Theta oscillations recorded by MEG and those recorded by the depth electrode are correlated without any phase delay. This figure is reproduced with permission from Korczyn et al. (2013).

Of particular significance for the current review, different coil configurations, for example the separation between gradiometers coils, will perform differently in terms of noise reduction (Figure 1D) and sensitivity to depth below the scalp (see also Figures 1–7 in Lopes da Silva, 2010). In general, axial gradiometers with one loop closer to the scalp and a second loop further from the scalp with a baseline of 3–8 cm are thought to provide optimum signal-to-noise ratios (SNRs) compared to magnetometers (the baseline of magnetometers can be regarded as infinite), due to the ability to reduce noise close to the system, yet minimize the suppression of brain activity further from the sensor. Planar gradiometers, with both coils overlying the scalp surface, achieve good rejection of environmental noise. However, they are constrained to have relatively short baselines (the baseline of commercial planar gradiometer systems is about 1.4–1.6 cm) and are therefore considered to have good SNRs for superficial brain sources, but less sensitivity to deep sources. In all cases, the sensitivity to deeper brain activity is relative to the amount of noise, including other brain activity. As a result, the impact of sensor design on depth sensitivity in MEG is often debated. It is generally thought that under ideal conditions, all sensor types may be able to detect deeper brain activity, with a slight advantage for axial gradiometers with appropriate baselines in presence of higher noise (Vrba and Robinson, 2001; Lopes da Silva, 2010).

## Modeling hippocampal activity in MEG—the inverse problem

From the measured data on the scalp, we typically wish to infer the spatiotemporal dynamics of neural activities at the source level, a process referred to as source localization. This is an ill-posed problem, because given a certain topography at the sensor level, there are an infinite number of configurations at the source level that could produce the measured magnetic fields (Baillet, 2017). However, by adding prior information and constraints, such as the anatomy from magnetic resonance imaging (MRI), and head geometry, this problem can be overcome by using source localization algorithms which model the magnetic fields that would be generated by a limited number of sources which best predict the measured data (Ioannides et al., 1990; Gorodnitsky et al., 1995; Im et al., 2005; Mattout et al., 2006; Wolters et al., 2006). To estimate sources from MEG scalp signals, the general procedure is to solve the forward and inverse problems sequentially (Hämäläinen et al., 1993; Attal et al., 2012). The forward solution computes the gain matrix composed of the contribution of each brain source to the external sensors, and with the head geometry modeled using realistic conductor models based on structural MRI (e.g., Hämäläinen and Sarvas, 1989; Fuchs et al., 1998; Nolte, 2003) or using more simplified spherical head models which assume the head can be well-modeled as a conducting sphere (e.g., Sarvas, 1987). Various inverse solutions are used to determine the current sources from the topographical pattern of activity seen in the data by considering the topographical patterns generated by forward solutions.

MEG inverse solutions can be roughly categorized into two classes. *Equivalent current dipole fitting* assumes relatively simple configurations of one or a few dipole-like sources which can be determined a priori from the observed fields and their location and strength parameters adjusted to fit the data. In contrast, *distributed-source imaging* methods do not make any assumptions about the number of active sources and therefore involve highly underdetermined solutions for many sources relative to the number of MEG sensors (Hämäläinen et al., 1993; Hämäläinen and Hari, 2002). Although dipole modeling of hippocampal activity as single or bilateral dipoles has been reported (e.g., Breier et al., 1998; Zouridakis et al., 1998), the assumption of simple source configurations is less likely to be valid in the case of the hippocampus, whose activities are more likely to overlap with those of neocortical sources. Consequently, distributed source imaging has been more commonly applied in recent studies (see Table 2).

**Table 2:** MEG simulation studies.

**Studies**	**Summary**
Chupin et al., 2002	Evaluated the relative contributions of hippocampal and neocortical regions to MEG signals. This paper focused on sensor level signals.
Stephen et al., 2005	Investigated whether MEG was able to differentiate between hippocampal activity and neocortical activity and between hippocampal activity and parahippocampal activity, in both sensor and source space. Source localization by dipole fitting.
Attal et al., 2007; Attal et al., 2012 (a review); Attal and Schwartz, 2013	Simulations were performed to determine the detectability of the activation from deep sources including the hippocampus. Performances of different depth weighted minimum norm inverse operators were compared.
Quraan et al., 2011; Mills et al., 2012	Investigated the ability of beamforming to localize hippocampal signals with different strengths in presence of different strengths of neocortical activation.
Meyer et al., 2017b	Used Bayesian model comparison to investigate which generative model (one containing cortical surface and one containing both cortical surface and the hippocampus) provided a more likely explanation of the dataset with simulated hippocampal activity. The performances of different inverse operators (multiple sparse priors, minimum norm, beamforming technique) were compared as well.

Distributed-source imaging methods can be subdivided into *non-adaptive* methods, including MNE (Hämäläinen and Ilmoniemi, 1984) and its variants such as low-resolution electromagnetic tomography (LORETA, Pascual-Marqui et al., 1994); and *adaptive* methods, such as beamforming (Robinson and Vrba, 1998; Gross et al., 2001; Hillebrand et al., 2005; Cheyne et al., 2007; Vrba et al., 2010) (see Sekihara et al., 2005 for a detailed comparison of adaptive and non-adaptive methods). As previously mentioned, MEG is conventionally assumed to be biased toward sources in superficial cortex because neuromagnetic signals decay strongly as a function of distance. Source localization algorithms which incorporate this assumption in their computations require additional corrections using depth weighting. Such weightings must be taken into consideration when considering the validity of MEG measures of hippocampal activity.

## MEG and the hippocampus: simulation studies

Simulation studies (Table 2) have been directed to two broad questions. First, can hippocampal activation be reliably detected by MEG sensors and if so, can this activity be dissociated from other signals and noise? Second, can hippocampal activation be localized by source localization algorithms, especially in the presence of concurrent sources in the neocortex?

An early study (Chupin et al., 2002) was carried out to evaluate the relative contribution of hippocampal and neocortical regions to MEG sensor signals from a forward problem point of view. This work simulated the activation of hippocampal and neocortical patches one after the other based on different current dipole moment densities estimated in those regions from animal models (Okada et al., 1996; Murakami and Okada, 2006). The external envelopes of the hippocampi were manually extracted from individual MRI images, while the neocortex was automatically segmented using Anatomist software (http://brainvisa.info/web/index.html). The MEG gain matrices (MEG forward model) for the hippocampus and neocortex were computed in a spherical head model in accordance with a CTF whole-head 151-channel system configuration (with first-order axial gradiometers). Results showed that average cortical activation increased linearly as a function of patch size, whereas hippocampal fields reached a plateau (saturation) for patches greater than about 2 cm^2^. This is likely due to the geometry of the hippocampus causing partial cancellation of the signal, similar to that seen during activation of large areas of the folded neocortex (Ahlfors et al., 2010). The neocortical magnetic fields were larger than the hippocampal fields, but the hippocampal fields (a mean of about 100 fT) were significantly larger than intrinsic MEG device noise level (10 fT at 10 Hz) and averaged brain background activity (a few tens of fT). These results show that although the hippocampus is farther away from the sensors relative to the neocortex, physiologically reasonable activations can result in magnetic fields large enough to be detected by MEG sensors. It may be that the higher current densities in the hippocampus relative to the neocortex compensate for the greater distance away from MEG sensors (Attal et al., 2007).

Stephen et al. (2005) explored whether MEG is able to differentiate between activity in different subfields of the hippocampus and superficial neocortex and between activity in the hippocampus and the parahippocampus, when activated sequentially and concurrently. Neocortical areas were segmented automatically using MRIVIEW software (Ranken et al., 2002). Due to the limited resolution of the anatomical MRI scan at 1.5T, the hippocampus was manually segmented. The anatomical model of the surface of the hippocampus included the dominant fields of the hippocampus including dentate gyrus, CA3, and CA1 as well as entorhinal cortex and presubiculum. The entire region for each subdivision of hippocampus was activated. The simulated signals were embedded in real background brain activities recorded using a 122-channel Elekta system (with planar gradiometers) from five epileptic patients in the resting state. They found that at the sensor level, the addition of real background brain activity to the simulated activity could significantly change the waveform of the simulated activity relative to that modeled without background activity. Activation of the hippocampus with one subfield or all subfields could be differentiated from activation from superficial neocortex and doubly dissociated. Parahippocampal activation could be differentiated from hippocampal activation when the two regions were simulated sequentially. Simultaneous activation of parahippocampus and hippocampus could also be differentiated from single hippocampal or parahippocampal activation in isolation, but no double dissociation was achieved in either case.

In source space, dipole fitting was used for source localization. To avoid biasing the results with known source locations, and to ensure the global minimum was reached, searches with random starting parameters were carried out across the whole brain. Results demonstrated that hippocampal sources and superficial cortical sources could both be located and differentiated, with 73% of all sources within a 10 mm error range and the mean amplitude-peak time difference between modeled peak and the simulated peak being 1.1 ms. When all the subfields of the hippocampus and dentate gyrus were simulated simultaneously, there was partial cancellation. However, hippocampal sources could only be differentiated from parahippocampal sources when the two regions activated sequentially and could not be resolved when activation overlapped in time.

Detailed simulations of deep brain areas, such as the hippocampus, the amygdala, and thalamus were carried out by Attal et al. (Attal et al., 2007, 2012; Attal and Schwartz, 2013) based on realistic anatomical and electrophysiological models to explore the detectability by MEG for these deep sources, and to compare the performance of different depth weighted minimum norm inverse operators (one depth weighted MNE and two noise-normalized depth weighted MNE algorithms). Unlike dipole fitting procedures, no prior of the activated source is needed for depth weighted minimum norm. As in Chupin et al. (2002), to mimic the activations of different areas, different values of current dipole moment density in different regions of interest were based on animal models to calculate the simulated MEG fields on a 151-channel sensor array (axial gradiometers) for each region of interest. The anatomical model corresponding to the source space was computed using individual MRI images at 3T. The segmentation of the cortical sheet and deep sources was done using the BrainVisa/Anatomist software (http://brainvisa.info/web/index.html). The activity at each location was modeled as an equivalent current dipole. Orientations of the current dipoles at neocortical areas were constrained to the local normal of the cortical mantle at each vertex location of the gray-white matter interface. Current dipoles were placed randomly in the inner volume grid in the thalamus, striatum, and the amygdala because the cells in those structures do not have preferred orientation. The hippocampus was modeled as the external envelope due to the difficulty of differentiating the precise inner structure based on 3T MRI images. The current dipoles were placed perpendicular to the local surface.

Simulation of activation from each region of interest in seven participants was performed for patch sizes ranging from 1 to 5 cm^2^ for surface patches and 1–5 cm^3^ for volume patches sequentially or concurrently. As expected, the simulated MEG fields for subcortical areas were 10 times lower than that for neocortex, but were strong enough to overlap parts of the distribution of the MEG field of neocortex, especially for the hippocampus and the amygdala. Then, the simulated fields were added to individual resting state MEG data, which were then inverted with a forward spherical head model and three inverse operators [depth weighted MNE (wMNE), dynamic statistical parametric mapping (dSPM) and standardized low-resolution electromagnetic tomography (sLORETA)], to localize the sources. DLE_g_ (the Euclidian distance of a solution's gravity center from the reconstructed source location to the true location) and DLE_m_ (the Euclidian distance of a solution's maximum from the reconstructed source location to the true location) were to assess the ability of the three inverse operators.

With a single subcortical activation, DLE_g_ was lower using wMNE than the two noise-normalized depth-weighted MEG inverse operators (dSPM and sLORETA) with errors < 8 mm in the majority of the hippocampus and the amygdala. Conversely, better DLE_m_ was obtained by dSPM and sLORETA, but the spatial patterns for the two inverse operators were not the same. sLORETA had a lower DLE_m_ in the deeper central regions, such as the thalamus, whereas dSPM had a very good estimation over the hippocampus but strong errors in the thalamus. Interestingly, activations of large patches give stronger DLEg, whereas large patches of 4 and 5 cm^2^ produce the strongest currents. This might be due to the cancellation of oppositely-directed currents as described above. For concurrent activation of two sources, one in the hippocampus and one in the visual cortex, when the two activations had little overlap (25%), hippocampal generators were well-estimated by the three inverse operators, but only dSPM maintained the local maximum in the hippocampus. With increasing overlap of the activation of the two areas, the performance of all of the methods decreased. wMNE had good detection of hippocampal activation when the overlap was up to 50%, whereas sLORETA and sSPM had good estimation of hippocampal activation even when the two sources were simultaneously activated, but created a local maximum in the thalamus (a deep ghost source).

The authors also computed point-spread functions to quantify the distortion of the source reconstruction by the inverse operators, namely, the spread of hippocampal sources to other cortical and subcortical areas. The resulting point-spread function maps of hippocampal sources showed that highest values were localized in the lateral and medial (parahippocampus and entorhinal cortex) and temporal lobe. Compared to wMNE point-spread function map, sLORETA point-spread function map of hippocampal sources showed a significant decrease in values in the neocortex but still significant values in the parahippocampal areas; however, the deeper regions in the thalamus and the nearest amygdala part showed a point-spread function value increase. dSPM point-spread function map of hippocampal sources showed small values in the neocortex and other subcortical structures. To quantify the distortion that is induced from other source locations, cross-talk functions were computed. No significant difference was found in the cross-talk function maps for the three inverse operators and the strongest values of the cross-talk function maps were located in the lateral temporal lobe, especially in the superior temporal sulcus, which suggests that activity in this region is most likely to influence the reconstructed hippocampal sources.

Simulation studies were carried out by Quraan et al. (2011) to estimate the ability of beamformers to localize hippocampal activation. In one set of simulations, dipole sources were placed in the anterior part of bilateral hippocampi based on MRI scans from 15 healthy adults. These source activities were simulated as a 50 ms segment of a 10 Hz sinusoid with a physiologically realistic range of amplitudes ranging from 10 to 40 nAm. Uncorrelated Gaussian noise at typical levels in an MEG system was then added to the simulated MEG field. Group average results showed that beamforming was able to recover sources in the region of the hippocampus at all simulated strengths. In a second set of simulations, the simulated hippocampal activity was added to the raw data of the same subjects during visual stimulation acquired with a 151-channel CTF system (axial gradiometers), either temporally displaced from or overlapping with the onset of visual evoked responses. For non-overlapping activity, if the simulation strength was greater than or equal to 30 nAm, the simulated field was visible on the sensor level global field power plot; but disappeared when the simulation strength was at or below 20 nAm. Beamforming was able to localize the hippocampal activation at all simulated source strengths. However, when simulated hippocampal activity was temporally overlapping with visual evoked fields the hippocampal signal was no longer visible due to leakage of source activity from the visual sources. To remove leakage from the visual source, condition subtraction was used. That is, the source localization image of the experimental condition was subtracted from that of the control condition, which evoked similar basic sensory responses but not the same degree of hippocampal activation. Leakage was well-controlled in the group-averaged images. However, at the individual participant level, in the presence of both low and high brain noise, hippocampal activation could be detected in only 2 or 3 out of 15 participants even with condition subtraction. This might indicate that localization of hippocampal activity at the individual level in the presence of strong time-locked sensory responses, particularly in visual areas may be difficult to achieve.

In a follow-up study, Mills et al. (2012) compared the accuracy of localizing hippocampal activation using different subtraction methods: post-localization subtraction (used in Quraan et al., 2011), and pre-localization subtraction. Pre-localization was done by first subtracting the sensor data of the two conditions and beamforming was performed on the difference data to localize the source. In situations of hippocampal activation embedded in either low or high brain noise, pre-localization outperformed post-localization subtraction method in terms of source localization accuracy and the ability to detect weak hippocampal activation. Applying the pre-localization method to empirical data acquired with a 151-channel CTF system while participants were performing a transverse patterning task, which has been shown to activate the hippocampus using other imaging modalities (e.g., Driscoll, 2003; Meltzer et al., 2008). However, at the individual level, hippocampal activation could still only be detected in six out of 14 participants, vs. two participants using post-localization subtraction. As noted in the study, a main drawback of sensor data subtraction is that changes in head position are not accounted for and can introduce errors in localization accuracy. Thus ideally, the experimental and control conditions should be interleaved in one experimental run so that the head movement and MEG-MRI co-registration error is the same.

Recently, Meyer et al. (2017b) used Bayesian model comparison to examine MEG sensitivity to hippocampal activity. The segmentation of the cortical sheet and the hippocampus was done using Freesurfer software (https://surfer.nmr.mgh.harvard.edu/) based on individual MRIs at 3T. A single dipole perpendicular to the surface of the hippocampal curvature or cortical surface was simulated in either the hippocampus or the cortical areas with a 300 ms segment of a sinusoidal waveform of 20 Hz and a dipole moment of 20 nAm. Gaussian white noise was added to simulated MEG fields. Two different realistic forward models were compared, one which included both the cortical surface and the hippocampus and one which only included cortical surface using three different inverse methods, namely, MNE, empirical Bayes beamformer (EBB), and multiple sparse priors (MSP). Free energy (Friston et al., 2006) was used as an index to quantify the model evidence of a given forward model with a given inverse operator. Free energy rewards the model that fits the data appropriately and penalizes models that are overly-complex. The researchers hypothesized if the simulation was in the hippocampus, the combined model with cortical areas and the hippocampus would outperform the model with only cortical areas (and would return a higher free energy value), because if using the cortical model, one needed a more complex combination of cortical sources to fit the data equally well. Results showed that for all three inverse operators, the combined model had a higher free energy value than the cortical model, but only the free energy value obtained from EBB and MSP inverse operators reached significance.

The source images echoed these results. When the correct model was used, the source maps of EBB and MSP were accurate and focal. When the wrong model was used, the source maps of EBB and MSP showed an increase in spatial spread and decrease in accuracy of the peak location. MNE returned the most diffuse source map with the peak outside the hippocampus, but the general pattern was similar across the three inverse operators. An alternative measure—dipole localization error (DLE)—was concordant with the results using free energy values and the cortical model gave higher DLE values than the combined model.

Activity in a nearby medial cortical area, 2.14 mm from the hippocampus, was then modeled to see whether the model comparison would return false positive results. The two models did not return significantly different results. These researchers also tested the influence of SNRs and MEG-MRI co-registration error on the model comparison. It was found that the more co-registration error and noise are, the harder it is to differentiate the two models. Most interestingly, poor SNRs were less harmful to the ability to differentiate models than co-registration error. When the co-registration error was > 3 mm, accurate model comparisons could not be achieved, indicating that minimizing head motion, and improving the accuracy of MEG-MRI co-registration will be important for MEG studies of hippocampal activity.

The results of the simulation studies reviewed above confirm that magnetic fields emanating from the hippocampus can be detected by MEG sensors at the surface using both planar and axial gradiometers. Cancellation occurs when hippocampal subfields and dentate gyrus are activated together but is only partial. Whether the hippocampal magnetic fields are visible or not on the global field power plot depends on the relative magnitude of background brain “noise,” including whether there are strong magnetic fields simultaneously arising in other cortical areas (e.g., from visual cortex). However, even in cases where the signal is not visible at the sensor level, source localization algorithms can potentially localize hippocampal sources at the group level. Thus, SNRs at the sensor level may not reflect the ability to localize weak hippocampal sources (Meyer et al., 2017b).

A variety of source localization algorithms, including dipole fitting, depth-weighted MNE, and beamforming have been successfully used to localize hippocampal sources. Unfortunately, no studies have compared these different algorithms using the same data, thus the relative advantages of different inverse algorithms remains to be determined. The study of Meyer et al. (2017b) indicates that the MNE algorithm may be biased to the neocortical surface. More important, they illustrated that MEG-MRI co-registration errors strongly influence localization accuracy for hippocampal sources. Thus, the combination of the use of continuous head movement localization and compensation (Stolk et al., 2013) and group averaging (since the co-registration errors should not be systematic across participants) is recommended for localization of the hippocampal sources.

## MEG and the hippocampus: empirical studies

Hippocampal sources have been reported by a number of empirical MEG studies (Table 3). These studies (unlike simulation studies) do not have a known ground truth of hippocampal activation. In this case, we need to rely on agreement between independent measures of hippocampal activity from MEG studies and other techniques/methods (e.g., iEEG, fMRI, lesion studies, and animal studies) using the same/similar paradigm. Experimental paradigms used to elicit hippocampal activation in MEG studies include memory encoding (e.g., Crespo-Garcia et al., 2016), retrieval (e.g., Guderian and Duzel, 2005), and integration (e.g., Backus et al., 2016), spatial navigation (e.g., Cornwell et al., 2008; Kaplan et al., 2012; Pu et al., 2017), violation detection (e.g., Ioannides et al., 1995; Nishitani et al., 1999; Garrido et al., 2015), and transverse patterning (e.g., Moses et al., 2009) (for more experimental paradigms, please refer to Table 3). The empirical evidence indicates that MEG can not only detect hippocampal signal reliably, but also contribute to revealing the neural mechanism and timing of cognitive processes in both normal healthy participants and patients.

**Table 3:** Empirical MEG studies of the human hippocampus.

**Article**	**Task**	**MEG system**	**Forward model**	**Inverse model**
Backus et al., 2016	Memory integration	Whole-head system with 275 axial gradiometers	Single shell head model	Beamforming
Breier et al., 1998, 1999	Memory recognition Auditory verbal and non-verbal Memory	148 magnetometers	Spherical head model	Dipole fitting
Campo et al., 2005, 2012	Working memory	148 magnetometers	Spherical head model	Multiple sparse priors (MSP)
Cornwell et al., 2008, 2010, 2012, 2014	Spatial navigation	275 axial gradiometers	Spherical head model	Beamforming
Cousijn et al., 2015	Resting state	102 magnetometers and 204 planar gradiometers	Spherical head model	Beamforming and Independent component analysis (ICA)
Crespo-Garcia et al., 2016	Item-place encoding	148 magnetometers	Realistic anatomical and electrophysiological model	Beamforming
Engels et al., 2016	Resting state	102 magnetometers and 204 planar gradiometers	Spherical head model	Beamforming
Fuentemilla et al., 2014	Autobiographical and Semantic retrieval	275 axial gradiometers	Single shell head model	Beamforming
Guderian and Duzel, 2005; Guderian et al., 2009	Memory encoding Memory retrieval	148 magnetometers	Not reported	Minimum-norm current–density reconstruction
Hamada et al., 2004	Oddball task	80 axial gradiometers	Spherical head model	Dipole fitting
Hanlon et al., 2003, 2005, 2011	Transverse patterning	122 planar gradiometers; 275 axial gradiometers	Spherical head model; Not reported in the paper of 2011	Dipole fitting; standardized Low Resolution Electromagnetic Tomography (sLORETA)
Heusser et al., 2016	Sequence encoding	275 axial gradiometers	Single shell	Beamforming
Hopf et al., 2013	Transverse patterning	151 axial gradiometers	Not reported	Beamforming
Ioannides et al., 1995	Oddball task	7 second-order gradiometers	Spherical head model	Magnetic field tomography (MFT)
Kirsch et al., 2003	Eyebink conditioning	122 planar gradiometers	Not reported	Dipole fitting
Kaplan et al., 2012	Spatial navigation	275 axial gradiometers	Single shell head model	Beamforming
Leirer et al., 2010	Transverse patterning	148 magnetometers	Spherical head model	Dipole fitting
Garrido et al., 2015	Sequence violation	275 axial gradiometers	Single shell head model	Beamforming
Martin et al., 2007	Transverse patterning Oddball task	102 magnetometers and 204 planar gradiometers	Spherical head model	Dipole fitting
Moses et al., 2009	Transverse patterning	151 axial gradiometers	Not reported	Beamforming
Nishitani et al., 1998, 1999; Nishitani, 2003	Oddball task Emotional picture discrimination	122 planar gradiometers	Spherical head model	Dipole fitting
Papanicolaou et al., 2002	Memory retrieval	148 magnetometers	Spherical head model	Dipole fitting
Poch et al., 2011	Delayed match-to-sample task	275 axial gradiometers	Single-shell head model	Beamforming
Pu et al., 2017	Spatial navigation	160 axial gradiometers	Spherical head model	Beamforming
Riggs et al., 2009	Scene recognition	151 axial gradiometers	Not reported	Beamforming
Taylor et al., 2011, 2012	Working memory Face recognition	151 axial gradiometers	Spherical head model	Beamforming
Tesche et al., 1996; Tesche, 1997; Tesche and Karhu, 1999, 2000	Oddball task Mental calculation and picture viewing Sensorimotor integration Working memory	122 planar gradiometers	Single compartment boundary element conductor model	Signal-space projection (SSP)
Zouridakis et al., 1998	Word recognition	148 magnetometers	Spherical head model	Dipole fitting

Hippocampal low frequency theta oscillations during virtual navigation are of considerable interest because of the linkages to classic studies in rodents (e.g., Buzsaki et al., 1983; Fox et al., 1986; O'Keefe and Recce, 1993) showing that when animals are actively exploring the environment, there is a striking increase in theta power in the hippocampus. Theta oscillations are believed to provide a timing mechanism for place cell firing (O'Keefe and Recce, 1993; Colgin, 2016) and are thought to play an important role in learning (Burgess and O'Keefe, 2011; Buzsaki and Moser, 2013; Lever et al., 2014). Subsequently, iEEG studies (e.g., Ekstrom et al., 2005; Jacobs et al., 2009; Watrous et al., 2013; Vass et al., 2016; Aghajan et al., 2017; Bohbot et al., 2017) have reported a comparable low frequency theta power increase in the human hippocampus during virtual, real and mental navigation.

Using a whole-head MEG system with 275 first-order axial gradiometers, Cornwell et al. (2008) recorded neuromagnetic responses of the brain of normal healthy participants while they were performing a virtual version of Morris water maze task (Morris, 1984), which has been used extensively to elicit hippocampal theta oscillations in rodent studies (e.g., Olvera-Cortes et al., 2004, 2012). In the virtual water maze task, participants are required to find a hidden platform fixed in a goal location in hidden platform condition and to randomly swim in a control condition. Beamforming was used to localize hippocampal theta signals. It was found that hippocampal theta power in the hidden platform condition was significantly stronger than that in the random swimming condition, in agreement with what has been found by human iEEG studies and animal studies in a similar behavioral context. Using a similar experimental task and another MEG system (a whole-head MEG system with 160 first-order axial gradiometers), Pu et al. (2017) replicated Cornwell et al. (2008)'s result that there was stronger left hippocampal theta power during hidden platform condition than the random swimming control condition. The consistency of the results from the two studies using different MEG systems and with different cohorts of participants provide good evidence that hippocampal signals can be reliably detected by MEG and constructed by source localization algorithms.

By adding another training set to the task, Pu et al. (2017) further found that human right hippocampal theta power was modulated by environmental novelty and the strength of right hippocampal theta power during new environmental encoding was associated with path lengths in both new (*r* = −0.5) and familiar (*r* = −0.57) environments (Figure 2), arguing strongly that human hippocampal right hippocampal theta rhythms play an important role in environmental encoding to form a cognitive map of the space, as shown in animal studies (e.g., Penley et al., 2013). The association between hippocampal theta power and behavioral performance on spatial navigation task advances our understanding of the behavioral correlates of human hippocampal theta rhythms, which is an important goal of decades of studies in this field (see Ekstrom et al., 2014 for a review). Moreover, the association also provides further evidence that the reconstructed hippocampal theta signal from MEG data should not be artifactual.

Comparison of source reconstructed images of patients with the hippocampus removed to that of normal controls in a hippocampus-dependent task offers a way to evaluate the validity of using MEG to detect hippocampal signals in empirical experiments. In an auditory oddball paradigm (a deviant sound embedded in a series of standard sounds) shown with iEEG (Halgren et al., 1980) to activate the hippocampus, Ioannides et al. (1995) and Okada et al. (1983) successfully localized hippocampal activity using 7-channel MEG recordings and suggest that the hippocampus is crucial in online violation detection (Garrido et al., 2015). In addition, Ioannides et al. (1995) compared the source localization image of a patient with hippocampus and amygdala removed to that of normal participants. They found no activation in the hippocampus and amygdala complex in the MEG source image of the patient, while clear hippocampal activities were seen in normal participants responding to the deviant sound. In another study with a whole-brain MEG recording and an auditory oddball paradigm, Nishitani et al. (1999) compared both event-related magnetic fields (ERFs) at the sensor level and the source activity responding to the deviant sound of the patients before and after resection of the hippocampus. They reported that after hippocampus resection, M400 at the anterior temporal channels on the resected side disappeared, and at the source level the activity in the resected mesial temporal area was lost. However for the patient who underwent inferior lateral temporal resection, M400 at the sensor level and the activity in the mesial temporal lobe were intact. These findings support the contention that MEG is sensitive to hippocampal signals, and argue against the possibility that the reconstructed hippocampal signals are artifactual. The results from patients also indicate that MEG is not only useful in investigating the hippocampal function of normal healthy participants, but can also be used to investigate the pathophysiology related to the hippocampus. With a virtual Morris water maze (vMWM) task and MEG recording, Cornwell et al. (2010) reported that people with major depressive disorders (MDD) showed impaired navigation performance on the vMWM task and this behavioral impairment was related to decreased right hippocampal theta power during navigation. The MEG result of the abnormal hippocampal functioning of MDD populations provide further support to the idea that hippocampal dysfunction is a key component of pathophysiology of MDD.

Simultaneous iEEG and MEG recordings provide perhaps the strongest evidence that that MEG can reliably measure hippocampal activity. In an intensive reading task, with depth electrodes placed in the hippocampus of four patients with epilepsy, Dalal et al. (2013) simultaneously recorded MEG and iEEG data. Results showed that depth EEG in the theta frequency range (4–8 Hz) from the hippocampus was strongly correlated at zero lag with MEG sensor signals over the temporal lobe (Figure 3). In another study, with a whole-head MEG system with 248 magnetometers, MEG signals were acquired while participants were performing an associative memory task. Crespo-Garcia et al. (2016) found that the power of low frequency (2–3 Hz) oscillations in the mid-posterior hippocampi reconstructed by beamforming was significantly stronger than that in the pre-trial interval, and the increased hippocampal power was negatively correlated with subsequent memory accuracy, indicating that local suppression of low-frequency activity is essential for more efficient processing of detailed information. These results were corroborated by results from simultaneously recorded iEEG data. Note that the direction of correlation with behavioral performance is opposite to what has been found for higher frequency 4–8 Hz in other studies (e.g., Cornwell et al., 2008; Kaplan et al., 2012; Pu et al., 2017), suggesting that there might be different subsequent memory effects for lower and higher low frequency oscillations. Further studies are needed to clarify the functional difference of the two hippocampal low frequency rhythms. Nevertheless, these results suggest MEG is able to uncover nuances of the functions of human hippocampal rhythms.

Although this review has mainly focused on evaluating hippocampal MEG signals in cognitive experiments, another good piece of evidence for detectability of hippocampal signals with MEG came from a recent study by Hillebrand and his colleagues, which used MEG and iEEG recordings to measures epileptic seizures. Hillebrand et al. (2016) found that the time series of the virtual sensor in the hippocampi reconstructed by beamforming from the MEG sensor signals accurately matched the spike discharges identified in recordings from depth electrodes placed in hippocampi.

Findings from parallel MEG and fMRI provide further validation for using MEG to detect hippocampal signals. Although fMRI signals are blood oxygen level-dependent (BOLD) signals related to neuronal activities (Ogawa et al., 1990) and MEG directly measures the magnetic fields induced by neuronal activities, the origin of at least some of the signals of the two imaging modalities are likely to originate from comparable underlying physiological processes (i.e., post-synaptic current flow; Hall et al., 2014). Moreover, a number of studies (e.g., Singh et al., 2002; Brookes et al., 2005; Muthukumaraswamy and Singh, 2008) have shown a close spatial relationship between MEG-derived oscillatory power in multiple frequency bands with BOLD. Further, it was found that the use of fMRI based priors to solve the MEG inverse problem would return higher model evidence in a Bayesian framework for fMRI constrained MEG source reconstruction (Henson et al., 2010). All these lines of evidence support the contention that fMRI and MEG have some spatial concordance (Hall et al., 2014).

Following this logic, using parallel fMRI and MEG recordings during a virtual spatial navigation task, Kaplan et al. (2012) reported fMRI observed increased hippocampal activation during movement initiation periods vs. stationary periods. Constructing the time series of this location from MEG sensor data using beamforming revealed that there was a theta power increase during movement initiation periods (0–1 s, 0 s was the movement onset), supporting the idea that hippocampal theta supports volitional navigation. The results indicate that MEG can not only tell us where the hippocampal region is responsible for a certain cognitive process, but can also tell us the specific neural mechanism and timing of this process (Dalal et al., 2008; Moses et al., 2011). In another study of functional connectivity in the resting state (Cousijn et al., 2015), independent component analysis (ICA) was used to identify networks in resting state fMRI data and MEG theta band activity reconstructed by beamforming. ICA of MEG theta band activity and fMRI data identified similar left and right lateralized hippocampal networks. Moreover, the spatial patterns of regions coactivated with the hippocampal network for fMRI and MEG was found to be highly correlated (r = 0.54). Further analyses showed that intrahippocampal theta obtained from MEG was negatively correlated with hippocampal-prefrontal cortex coactivation obtained from fMRI. While the exact relationship between MEG and fMRI signals is a complicated topic (Hall et al., 2014) and beyond the scope of this paper, the point here is hippocampal activities reported by both fMRI and MEG argue strongly that hippocampal activities can be detected by MEG.

Important albeit indirect evidence that MEG is sensitive to deep source comes from dynamic causal modeling (DCM). Using simulation data, David et al. (2011) showed that DCM is able to differentiate data without hidden sources and the data where hidden sources were present. Based on this, they applied DCM to an empirical dataset of language processing to investigate whether there was an assumed hidden source (the thalamus, which was revealed by an iEEG study (Wahl et al., 2008) with a similar experimental paradigm) or not. They found that the model including the thalamus explained the data better than the model without the thalamus. In a recent effort, using MEG measurement, Garrido et al. (2015) reported more hippocampal theta and medial prefrontal theta activation during sequence violation detection. The sources were also documented in a previous fMRI study (Kumaran and Maguire, 2006) using the same experimental task. Further, using DCM, the models which support interactions between the medial prefrontal cortex and the hippocampus and the model which supports no interactions between the two sources were compared. It was found that the model in which prefrontal theta drives the hippocampal theta explained the data best, in line with the idea that the circuit between the prefrontal cortex and the hippocampus supports important cognitive processing (Yoon et al., 2008; Weilbacher and Gluth, 2016; Eichenbaum, 2017) and the idea that theta is critical in long-range information transformation (Miller, 1991; Buzsaki, 1996; Siapas et al., 2005; Benchenane et al., 2010). This idea has been corroborated by more recent MEG studies in various cognitive tasks (e.g., Kaplan et al., 2014; Backus et al., 2016) which have demonstrated that the hippocampus and the medial prefrontal cortex form a functional network through theta power or phase coupling between the two regions for information communication.

## Conclusions and future directions

Taken together, the evidence reviewed above strongly supports the contention that MEG can reliably detect signals from the hippocampus. We can draw on three converging lines of evidence:

1. Physiological considerations. The principal neurons of the hippocampus are uniformly aligned with their dendrites in parallel in the same direction perpendicular to the hippocampal surface (Lorente de No, 1947), such that the intracellular currents produced by synchronization of those neurons should be detectable by MEG (Murakami and Okada, 2006). In addition, the current dipole moment density in the hippocampus is larger than that in the neocortex (Okada et al., 1997), such that it can generate magnetic fields strong enough to be sensed by distant MEG sensors (Attal et al., 2007). Although the geometry of the hippocampal formation is folded, it can be shown that significant cancellation occurs only when all hippocampal subfields and dentate gyrus are activated simultaneously (Stephen et al., 2005).
2. Simulation studies. Simulations show that hippocampal signals can be sensed by MEG sensors even with different pick up coil configurations, such as axial vs. planar gradiometers. Various source localization algorithms can be used to reconstruct hippocampal sources from MEG data, as long as the algorithm can suppress the strong signals from other brain regions including the neocortex. Beamforming algorithms are effective in suppressing the signal outside the region of interest without compromising the signal from the region of interest. Compared to source image reconstructed by MNE, the source images reconstructed by beamforming were shown to be more focal and the peak was better localized to the hippocampus (Meyer et al., 2017b). Compared to dipole fitting or MSP, no priors about activation locations need to be specified for beamforming. However, it is important to consider appropriate control conditions during experimental design, to alleviate leakage at the group level, as well as other factors such as the number of trials to increase the amount of the data used for computing the covariance matrix (Brookes et al., 2008). Although the main focus here was on spatial accuracy of the reconstructed hippocampal signals, it is also worth noting that temporal accuracy of the reconstructed hippocampal signal is high. As shown in Stephen et al. (2005), the mean amplitude-peak time difference between modeled and simulated peaks was ~1 ms. Accurate temporal resolution is critical for reliability of connectivity analyses.
3. Empirical studies. A range of empirical studies have successfully shown that MEG can reliably detect and localize the hippocampal signals in various experimental paradigms which have already been shown hippocampal activation using other modalities and methods. Simultaneous iEEG and MEG recordings, and parallel MEG and fMRI studies both provide good evidence that MEG is capable of detecting hippocampal signals. The finding that hippocampal signals could not be extracted from MEG data in a patient after resection of the hippocampus and amygdala complex supports the conclusion that hippocampal signals reconstructed in normal participants are not artifactual (Ioannides et al., 1995; Nishitani et al., 1999).

Currently, empirical MEG studies of the hippocampus still heavily rely on group averages. It is still challenging to study hippocampal function using MEG at the individual level, an important step for studies of individual differences and clinical implications. As shown in the simulation study of Meyer et al. (2017b), at the individual level, if the co-registration error was > 3 mm, model comparison could not choose the correct model. This problem was compensated with subject-specific headcasts using 3D printing recently introduced to the MEG community (Meyer et al., 2017a). Although the latter approach is likely not feasible or cost-effective for general application, taking care to minimize head motion will significantly improve the sensitivity of MEG to hippocampal signals. Moreover, accurately detecting the hippocampal signal also depends on accurate forward modeling, although it still needs to validate the realistic model based on individual MRI segmentations (Dalal et al., 2013). Moreover, not every MEG toolbox has implemented realistic head modeling and it is still computationally expensive to construct a realistic forward model. Nonetheless evidence to date suggests that the accuracy of reconstructed hippocampal signals will be improved if the hippocampal subfields are modeled based on the realistic electrophysiological properties. To this end, acquiring individual MRI images using ultra-high field MRI system at 7 or more tesla would facilitate more accurate segmentation of the hippocampus and more accurate forward modeling. This will be a significant step in the effort to improve the differentiability of MEG signals in hippocampal subfields. Of course, more efficient computational approaches in realistic forward models are important for future advances and implementation. This in combination with decreased head motion and modeling error can further improve the spatial resolution of the reconstructed MEG signal at the level of hippocampal subfields. Improved spatial resolution and a decrease in the cross talk among the reconstructed signals will also advance the capabilities of connectivity analysis for investigating interactions between the hippocampus and the neocortex.

Box 1 summarizes the crucial factors to be considered for an MEG experiment aiming at studying the hippocampal function.

Box 1 **Critical factors in using MEG to study hippocampal function.**

Hardware considerations. All types of MEG sensors are able to detect weaker deeper brain activity. However, in presence of higher noise (including activities from other brain areas), MEG systems with axial gradiometers with appropriate baselines may have a slight advantage to detect hippocampal activity (Vrba and Robinson, 2001; Lopes da Silva, 2010). The next generation of OPM-based MEG systems offer new possibilities for interrogating hippocampal function with SNRs three to four times greater than the current state-of-the-art.

Forward modeling. Simulation and empirical studies have shown that both analytical (spherical head models) and numerical (realistic head models) forward models can be used to reliably localize hippocampal activities. In principle, the more accurate the forward modeling is, the more accurate the reconstructed source activities will be.

Inverse operators. A variety of inverse operators have been successfully used to image the hippocampus, provided methods are used to suppress stronger signals from other brain regions. Beamforming is good at suppressing the activity outside the region of interest without compromising the signal from the region of interest. If the interest is in hippocampal rhythms, SAM (Robinson and Vrba, 1998), LCMV (Van Veen et al., 1997) or DICS (Gross et al., 2001) beamformers should be employed. Event-related magnetic fields from the hippocampus can be studied with event-related beamforming methods that utilize signal averaging with advantages over dipole modeling (Cheyne et al., 2007). Weighted minimum-norm methods are recommended to avoid bias toward superficial sources (Attal et al., 2007).

Finally, although this review focuses on current MEG methodology, it should be noted that recent hardware advances such as the introduction of new types of MEG sensors may further improve MEG measures of deep sources. For example, optically pumped magnetometers (OPMs; Boto et al., 2017) although having poorer sensitivity than conventional SQUID sensors, do not require cooling with liquid helium and can thus be brought much closer to the surface of the head than conventional SQUID-based sensors, resulting in a three-to four-fold increase in SNR; and it also allows movement and thus opens up new possibilities of studying hippocampal rhythms during real navigation and scanning populations like children and patients (Boto et al., 2018). The evidence of the current review confirms that conventional SQUID MEG systems are capable of interrogating human hippocampal function; although still in early stages of development, OPM-based MEG systems promise significantly better sensitivity deep source activity by substantially reducing the distance between the hippocampus and sensors (Borna et al., 2017).

To conclude, we have reviewed evidence indicating that MEG is capable of detecting hippocampal signals reliably and provides sensitive and behaviorally-relevant measures of hippocampal functions across a variety of experimental paradigms in both normal healthy participants and patients. It opens a unique window for us to study the functional and behavioral correlates of hippocampal rhythms and the neurophysiological mechanisms of inter-regional connection between the hippocampus and other brain regions without depending on the limited opportunities for recordings from pre-surgical patients. This advantage will thus allow us to bridge the gap between animal studies and human hippocampal studies and between the computational models and human neurophysiological data in routine experimentations.

## Author contributions

YP conducted the review; YP, DC, BC, and BJ wrote the paper.

### Conflict of interest statement

The authors declare that the research was conducted in the absence of any commercial or financial relationships that could be construed as a potential conflict of interest.

## Abbreviations

CA: Cornu ammonis
CSF: Cerebrospinal fluid
DICS: Dynamic imaging of coherent sources
DLE: Dipole localization error
EBB: Empirical Bayes beamformer
ECoG: Electrocorticography
EEG: Electroencephalography/Electroencephalographic
ICA: Independent component analysis
iEEG: Intracranial electroencephalography/electroencephalographic
LCMV: Linearly constrained minimum-variance
MEG: Magenetoencephalography/Magnetoencephalographic
MNE: Minimum norm estimation
MRI: Magnetic resonance imaging
MSP: Multiple sparse priors
MSR: Magnetically shielded room
OPM: Optically pumped magnetometer
SAM: Synthetic aperture magnetometry
SNR: Signal-to-noise ratio
SQUID: Superconducting quantum interference device
SSP: Signal-space projection
dSPM: Dynamic statistical parametric mapping
sLORETA: Standardized low-resolution electromagnetic tomography
SQUID: Superconducting quantum interference device
vMWM: virtual Morris water maze.
